# Differences between subjective experiences and observed behaviors in near-fatal suicide attempters with untreated schizophrenia: a qualitative pilot study

**DOI:** 10.1186/s12991-015-0055-1

**Published:** 2015-04-15

**Authors:** Taiju Yamaguchi, Chiyo Fujii, Takahiro Nemoto, Naohisa Tsujino, Kiyoaki Takeshi, Masafumi Mizuno

**Affiliations:** Department of Neuropsychiatry, School of Medicine, Toho University, 6-11-1 Omori-nishi, Ota-ku, Tokyo 143-8541 Japan; Department of Forensic Psychiatry, National Center of Neurology and Psychiatry, National Institute of Mental Health, 4-1-1 Ogawa-Higashi, Kodaira, Tokyo 1878553 Japan

**Keywords:** Untreated schizophrenia, Suicide, Help-seeking, Family, Psychoeducation

## Abstract

**Background:**

In cases of untreated schizophrenia, the patients’ entourage often does not recognize the psychotic symptoms of the patient and the possibility that the patient may attempt suicide. The aim of this study was to investigate the discrepancies between the subjective experiences and observed behaviors in near-fatal suicide attempters with untreated schizophrenia.

**Methods:**

A semi-structured interview was carried out with seven near-fatal suicide attempters with untreated schizophrenia to examine the subjective experiences at the time of the suicide attempt. The families of the patients were also interviewed to determine their recognition of the patients’ psychotic symptoms and the suicidal ideation. The interview data were analyzed qualitatively.

**Results:**

Six subjects were undergoing exacerbation of the psychotic symptoms at the time of exhibiting the suicide-related ideation. One subject had been in a prolonged depressive state before attempting suicide. Although all the patients experienced severe distress due to psychotic symptoms and depressive mood, they all exhibited only low level or no help-seeking behavior, and six of seven families had not recognized the change in the patient’s mental condition.

**Conclusions:**

Appropriate information about schizophrenia should be provided to the general public so that any help-seeking by the patients with this disease is not overlooked. In addition, accessible early intervention services for psychosis should be established.

## Background

Suicide is one of the most significant causes of unexpected death among patients with schizophrenia, particularly during the early phase of the illness [1-4]. Given that 10%–28% of patients diagnosed with a first episode of psychosis have already attempted suicide prior to the first treatment [5-7] and that the risk of suicide is high even in the prodromal phase of schizophrenia [8], there is a possibility that a considerable number of patients commit suicide prior to being recognized as suffering from schizophrenia needing appropriate treatment. This suggests that the actual risk of suicide in schizophrenia might be higher than the estimated based on epidemiological studies. Moreover, previous studies indicate that the longer the duration of untreated psychosis (DUP), the higher the risk of suicide-related behaviors [9-11]. Thus, there is no doubt that a clear understanding of the association between the characteristics of the clinical features and suicide-related behaviors in untreated schizophrenia is critical to provide necessary care to those with who are at a high risk of suicide arising from treatable symptoms.

While depressed mood, low self-esteem, hopelessness, and previous suicide attempts are well-established risk factors [12,13], the relationship between the psychiatric symptomatology and suicide in people with schizophrenia is not yet well understood [12,14]. In order to examine the causes and psychological processes of suicide, the psychological autopsy method has been widely used in scientific research [15]. Although psychological autopsy is a well-established method, its efficiency is limited in cases of untreated schizophrenia, because the patient’s entourage had often not recognized the psychotic symptoms of the patient [16,17], and as a result, they may not be able to report on the mental status associated with the suicide-related behavior.

Therefore, instead of psychological autopsy, we conducted semi-structured interviews of persons diagnosed with schizophrenia for the first time in their lives only after they exhibited near-fatal suicide attempts [18] in order to examine the subjective experiences which are potentially relevant to suicide in people with untreated schizophrenia. We also conducted interviews with patients’ family members to investigate the families’ awareness of the mental state of these persons prior to their suicide attempts. A better understanding of the subjective experiences related to suicide and of the discrepancies between the subjective experiences and observed behaviors may be helpful to devise more effective measures for preventing suicide among persons with schizophrenia.

In this article, the terms to describe suicidality were followed the guidelines published by Silverman et al. [19,20].

## Methods

### Study subjects

Patients were included in the study if they met the following criteria: admission to the emergency care unit of the Toho University Omori Medical Center in Tokyo between January 2007 and June 2011, after a near-fatal suicide attempt [16] and consulting the psychiatric department; aged 18 to 65 years; diagnosis of schizophrenia (F20 according to the ICD-10 research criteria; World Health Organization, 1992); no neurological or endocrine disorders to explain the psychosis; no mental retardation; no substance-induced psychotic disorders; no previous history of psychiatric consultation and/or treatment; willing and able to give informed consent. Seven patients met the criteria during the study period.

### Method

Semi-structured interviews of patients fulfilling the above criteria were conducted just after the psychiatrists responsible for the patients decided that the patient’s physical and mental conditions was stable enough to be interviewed. On the same day, semi-structured interviews of the patient’s family members were also conducted separately from the patients. Family members gave written informed consent to participate in the study. The topics covered our semi-structured interviews were as follows.

Interview with the patient:

- Subjective experience of symptoms,
- Mental state at the time of the suicide attempt,
- Psychosocial stressors,
- History of previous suicide attempts,
- Timing of appearance of vague suicidal ideation,
- Timing of appearance of definite suicidal ideation, and
- Help-seeking behaviors.

Interview with the family:

- Objective observations,
- Recognition of the patient’s mental health problems (possibility that the patient has psychosis),
- Recognition of the patient’s suicidal ideation, and
- Realization of the patient’s need for psychiatric consultation.

All the interviews were conducted by TY. Using a grounded-theory approach [21], TY, CF, and MM coded the transcriptions of the recorded data and analyzed by a series of discussions to identify the subjects’ clinical characteristics, subjective experiences at the time of the suicide attempt, psychosocial stressors related to suicide-related ideation, psychological processes leading to the suicide attempt, characteristics of the patients’ help-seeking behaviors, and the families’ recognition of the patients’ psychiatric problems before their suicide attempts.

Ethical approval for the research was obtained from the Ethical Research Committee of Toho University Omori Medical Center.

## Results

### Clinical manifestations and subjective experiences

Cases 1–6 showed rapid exacerbation of psychotic symptoms preceding the suicide attempt. Case 5 recalled that the death of his aunt may have been the trigger of exacerbation of the symptoms. Case 6 said that psychotic symptoms became more pronounced after she was hospitalized for physical illness. Cases 1–4 could not recall any trigger for the exacerbation of the symptoms. Case 7 was in a severe depressed mood but had no acute deterioration of psychotic symptoms preceding the suicide attempt.

The results presented in Table 1 summarize the subjective experiences of the patients. In regard to the contents of the hallucinations, cases 1 and 4 had command hallucinations; case 1 followed a command to exhibit suicide-related behavior; his behavior was not suicide-related in the actual sense, as he did not show suicide-related ideation at any time during the entire process. He simply followed the voices he heard suggesting that said that he would be able to fly if he jumped from the sixth floor of his apartment building, and he had not expected that he would fall to the ground. Case 4 attempted suicide because of the severe distress caused by the powerful hallucination saying ‘drop dead!’ not because he followed the command hallucination.

**Table 1:** **Characteristics of patients and their subjective experiences**

**Case number**	**Gender**	**Age**	**DUP (months)**	**Marital status**	**Length of formal education**	**Employment status**	**Dominant clinical symptoms**	**Timing of the appearance of vague suicide-related ideation**	**Timing of the appearance of definite suicide-related ideation**
1	Male	40	156	Single	16	Unemployed	*Command hallucination saying ‘jump off’*	(−)	(−)
							Delusion of persecution, delusion of reference		
							Thought broadcasting		
							Insomnia, social avoidance		
	Male	34	20	Single	12	Unemployed	*Delusion of persecution, delusion of reference*	4 days before	Just before
							Acute stress reaction		
							Delusion of observation		
							Thought broadcasting		
							Depressed mood as a reaction to the psychotic episode		
							Insomnia, social avoidance		
3	Male	30		Single	14		*Auditory hallucination*	Just before	
							Acute stress reaction		
							Delusion of persecution, delusion of reference		
							Depressed mood as a reaction to the psychotic episode		
							Insomnia, Social avoidance		
4		26	3	Single	14	Unemployed	*Command hallucination saying ‘drop dead’*	10 days before	Just before
							Acute stress reaction		
							Delusion of persecution, delusion of reference		
							Thought broadcasting		
							Depressed mood as a reaction to the psychotic episode		
							Insomnia		
	Male	55	300	Single	16	Unemployed	*Delusion of persecution, delusion of reference*	6 hours before	6 hours before
							Acute stress reaction		
							Depressed mood as a reaction to the psychotic episode		
							Social avoidance		
6	Female	59		Married	14		*Delusion of reference*	3 hours before	
							Acute stress reaction		
							Depressed mood as a reaction to the psychotic episode		
							Social avoidance		
7	Male		22	Divorced		Unemployed	*Depressed mood*		11 days before
							Hopelessness		
							Insomnia, loss of appetite		
							Hypobulia		
							Delusion of reference, Thought broadcasting		
							Social avoidance		

Italicized data are the most influential symptoms on each case’s suicidal behavior.

In cases 2–6, the rapid exacerbation of positive symptoms (auditory hallucinations, delusion of persecution, and so forth) inflicted such unbearably painful experiences that they felt a sudden surge of suicide-related ideation. In these cases, the period between the appearance of definite suicide-related ideation and the suicide attempt was very short. None of cases 1–6 had attempted suicide previously.

In case 7, mainly depressed mood and hopelessness were associated with the suicide-related ideation. This patient had experienced a psychosocially stressful life event that had triggered the suicidal ideation. After his apartment building was recognized to be in need of remodeling, his depressed mood deteriorated because he did not have enough money to have the building remodeled. Case 7 had also attempted suicide 11 days prior to the suicide attempt that resulted in hospitalization. In the previous attempt, he had changed his mind about suicide and stopped the behavior of his own will. In this subject, the period from the appearance of definite suicidal ideation to the suicide attempt was longer than in the other cases. He said that he was fluctuating between wanting to die and wanting to live until the near-fatal suicide attempt.

### Help-seeking behaviors and recognition by the families

Table 2 shows the patients’ help-seeking behaviors and recognition of the symptoms by the families. All of the patients exhibited only low level or no help-seeking behaviors in response to their psychotic symptoms and distress. Therefore, most family members did not recognize the patients’ need for psychiatric treatment.

**Table 2:** **Observed behaviors: patient’s help-seeking behavior and the families’ recognition**

	**Patient’s help-seeking behavior**	**Families’ recognition of the possibility that the patient has psychosis**	**Families’ realization that the patient may attempt suicide**
Case 1	None	(−)	(−)
Case 2	Not enough	(−)	(−)
Case 3	Not enough	(−)	(−)
Case 4	Not enough	(+)	(−)
Case 5	None	(−)	(−)
Case 6	Not enough	(−)	(−)
Case 7	Not enough	(−)	(−)

Case 2 told his mother only once that he felt like his conversations were wiretapped. His mother took little note of this because he was not talking in a very serious tone. Case 3 handed a note to his mother saying that he had heard something unusual, but the mother had not taken this seriously. Case 7 told his friends, but not his family, that he often felt depressed. In case 4, while the family had recognized that the patient needed to see a psychiatrist because of his restless behavior, they failed to persuade him to seek psychiatric treatment. Although case 4 himself recognized that he had psychiatric problems, he believed that no medical treatment could ameliorate his problems, and his family had no information about whom to consult in such a situation.

## Discussion

Most existing reports on the role of psychotic symptoms in suicidality have focused on the quantitative aspects of the symptoms. However, given that the relationships between psychotic symptoms and suicide-related ideation are diverse, the qualitative aspects of the symptoms should also be considered. Since the direct examination of the psychological processes of suicide completers with untreated schizophrenia is impossible, we examined the characteristics of the suicide-related behaviors of near-fatal suicide attempters with untreated schizophrenia in order to obtain a clearer understanding of the psychological processes leading to suicide. We also focused on the discrepancies between the subjective experiences and observed behaviors in people with schizophrenia in an attempt to devise more effective suicide prevention strategies.

Six out of seven patients in this study exhibited a depressed mental status at the time of the suicide attempt. This is to some extent in line with previous research indicating that suicide attempts during untreated psychosis are associated with depressive episodes [22]. However, the present results show that depressed mood itself does not always have a direct relation to suicide. Subjectively, case 7 had severe depressed mood induced by psychosocial factors that was directly related to suicide, whereas cases 2–6 suffered from relatively low-level depressed moods not directly related to the suicidal behavior. In the latter cases (cases 2–6), rapid exacerbation of the psychotic symptoms was associated with an increased risk of suicide.

In cases that experienced rapid exacerbation of the psychotic symptoms, the period between the appearance of definite suicidal ideation and the near-fatal suicide attempt was very short, and these patients attempted suicide without hesitation, unlike case 7, who did not show rapid exacerbation of the psychotic symptoms. In regard to case 1, who followed the direction of the command hallucination, his sudden suicide-related behavior can be interpreted as being a consequence of his thinking being dominated by the hallucination. In the other cases (cases 2–6), patients’ minds were not entirely dominated by the delusions or hallucinations, but the rapid exacerbation of psychotic symptoms caused an acute stress reaction in the patients. For people with psychotic symptoms, delusions of persecution and auditory hallucinations can be challenging or traumatic life events that may cause social avoidance, depression, or PTSD [23-25]. The results of the present study suggest that rapid exacerbation of psychotic symptoms can cause an acute stress reaction leading to fatal suicidal behavior. This suggests that crisis intervention in cases with rapid exacerbation of the psychotic symptoms is vital to prevent suicide, even if the patient does not have suicide-related ideation and/or severe depressed mood at the time.

These findings seem to be inconsistent with the results of previous research showing that suicide-related behavior is not common at the peak of psychosis [26,27]. Whether delusional ideas and/or hallucinations function as protective factors or risk factors for suicide may depend on the contents [28] and/or intensity of such symptoms. In regard to command hallucinations, which have been recognized as risk factors for suicide in patients with psychosis [29], it is important for suicide prevention to consider the contents of the hallucinations and how these symptoms affect each patient. In this study, one case responded to the command hallucination, resulting in self-injurious behavior (case 1), another case developed acute stress reaction due to the contents of the powerful command hallucinations that were quite distressing to the patient (case 4).

The results of the present study demonstrate that there are several obstacles to provide early interventions in response to rapid deterioration of psychotic symptoms: poor public awareness and failure to recognize the symptoms of psychosis, lack of appropriate help-seeking behaviors from the patients for distressing symptoms, and limited social resources for mental health services.

Previous studies suggested that families and caregivers play a principal role in shortening the delays in providing treatment for psychosis [30,31]. However, public knowledge of mental disorders, and in particular, of specific schizophrenia symptoms, is poor [32], and families tend to underestimate patients’ early psychotic symptoms [31]. In cases 2 and 3, despite the families knowing that the patients suffered from delusions of persecution or auditory hallucinations, they never believed that the patients might be in need of psychiatric treatment.

In Japan, local authorities have started setting up educational activities for preventing suicide based on the Suicide Prevention Law enacted in 2006. Most of these are educational programs on depression targeted at gatekeepers [33,34]. Previous studies have shown that educating gatekeepers on depression decreased suicide rate [35,36]. On the other hand, the results of the present study indicate that most family members did not recognize the signs of psychosis in the patients, suggesting that the number of people with untreated schizophrenia committing suicide may be higher than suggested by the statistics. Given the epidemiological data that schizophrenia occurs mostly in adolescents [37,38] and that the mean DUP in Japan is 13.7–20.3 months [39,40], proactive public information campaigns about psychosis and incorporating mental health literacy education in schools as part of the curriculum are necessary for devising more effective suicide prevention strategies, especially for the younger generation.

Considering the results of this study and previous research [41], we acknowledge the fact that appropriate help-seeking is often difficult for people with psychotic symptoms. Recent findings indicate that educating the public about the early signs and symptoms of psychosis and schizophrenia will reduce both the DUP and the suicide-related behaviors [42,43]. These educational activities are expected to foster help-seeking behaviors in people suffering from psychotic symptoms.

In case 4, despite the family recognizing the need for psychiatric treatment for the patient, the lack of a community-based psychiatric crisis intervention system prevented the implementation of a well-timed intervention. Recently, several institutions have made efforts to promote early psychiatric interventions in Japan [44,45]. However, mental health services for those who refuse to see a psychiatrist (case 4) or people with social avoidance (cases 1–3 and 5–7) are still extremely limited. The establishment of a system for early intervention in patients with psychosis, including outreach services, is urgently needed to promote suicide prevention.

This study had several limitations. First, since the sample size was very small, our study sample cannot be considered as representative of ‘suicide in untreated schizophrenia in general’. Second, there was no control group matched with the cases. Finally, the possibility of incomplete and biased information cannot be excluded, because the data were collected retrospectively. Nonetheless, this is the first qualitative study to examine the psychological processes leading to suicide in persons with untreated schizophrenia and the discrepancies between the subjective experiences and observed behaviors of these persons. We expect that our findings will contribute to the development of more effective suicide prevention strategies.

## Conclusions

In people with schizophrenia, which is mainly characterized by symptoms of hallucinations and delusions, there is the possibility that suicide ideations rapidly progress to suicide attempts. Therefore, it is essential to establish a psychiatric crisis intervention system in the community for providing timely and appropriate professional intervention to prevent suicide. The general public (including those who impart education) should be given information not only on depression and stress-related disorders but also on schizophrenia so that help-seeking by the patients is not overlooked.
